# Radiological manifestations and clinical findings of patients with oncologic and osteoporotic medication-related osteonecrosis of the jaw

**DOI:** 10.1038/s41598-024-59500-x

**Published:** 2024-04-16

**Authors:** Jeong Won Shin, Jo-Eun Kim, Kyung-Hoe Huh, Won-Jin Yi, Min-Suk Heo, Sam-Sun Lee, Soon-Chul Choi

**Affiliations:** 1 Department of Orthodontics, Institute of Oral Health Science, Ajou University School of Medicine, Suwon, Republic of Korea; 2 Department of Oral and Maxillofacial Radiology and Dental Research Institute, School of Dentistry, Seoul National University, Seoul, Republic of Korea; 3 Department of Oral and Maxillofacial Radiology, School of Dentistry, Seoul National University, Seoul, Republic of Korea

**Keywords:** Medication-related osteonecrosis of the jaw (MRONJ), Osteoporosis, Malignancy, Clinical assessment, Radiographic assessment, Diseases, Medical research, Risk factors, Signs and symptoms

## Abstract

Medication-related osteonecrosis of the jaw (MRONJ) poses a challenging form of osteomyelitis in patients undergoing antiresorptive therapies in contrast to conventional osteomyelitis. This study aimed to compare the clinical and radiological features of MRONJ between patients receiving low-dose medications for osteoporosis and those receiving high-dose medications for oncologic purposes. The clinical, panoramic radiographic, and computed tomography data of 159 patients with MRONJ (osteoporotic group, n = 120; oncologic group, n = 39) who developed the condition after using antiresorptive medications for the management of osteoporosis or bone malignancy were analyzed. The osteoporotic group was older (75.8 vs. 60.4 years, *p* < 0.01) and had a longer duration of medication usage than the oncologic group (58.1 vs. 28.0 months, *p* < 0.01). Pus discharge and swelling were more common in the osteoporotic group (*p* < 0.05), whereas bone exposure was more frequent in the oncologic group (*p* < 0.01). The mandibular cortical index (MCI) in panoramic radiographs was higher in the osteoporotic group (*p* < 0.01). The mean sequestra size was larger in the oncologic group than in the osteoporotic group (15.3 vs. 10.6 mm, *p* < 0.05). The cured rate was significantly higher in the osteoporotic group (66.3% vs. 33.3%, *p* < 0.01). Oncologic MRONJ exhibited distinct clinical findings including rapid disease onset, fewer purulent signs, and lower cure rates than osteoporotic MRONJ. Radiological features such as sequestrum size on CT scan, and MCI values on panoramic radiographs, may aid in differentiating MRONJ in osteoporotic and oncologic patients.

## Introduction

Medication-related osteonecrosis of the jaw (MRONJ), also known as bisphosphonate (BP)-related osteonecrosis of the jaw (BRONJ) or antiresorptive-related osteonecrosis of the jaw, is a potentially severe side effect of some medications^1,2^. BPs is representative antiresorptive drugs that cause MRONJ, with a potent chemical affinity for bones. These drugs specifically inhibit osteoclastic activity and are used to manage skeletal-related events (SREs) or lytic lesions^3,4^. Therefore, they are used widely to treat osteoporosis, multiple myelomas, and bone metastasis, preventing metastatic osteolysis observed in breast, prostate, and lung cancers^4^. In addition to BPs, drugs such as denosumab, RANKL inhibitors, and antiangiogenic agents have been identified as related drugs^1–3^. Since the first BRONJ cases described by Marx in 2003 and Ruggiero in 2004, numerous cases have been reported in patients who received antiresorptive medications caused by conditions such as bone metastatic tumor or osteoporosis. The AAOMS updates the staging and treatment plans for MRONJ through continuous position papers^3^.

The reported prevalence of MRONJ is 1–3% among patients with oncologic conditions who received high-dose antiresorptive therapy and 100 times less among patients with osteoporosis who received lower-dose therapy^1,3,5^. General advances in cancer care have increased the possibility of prolonged overall survival in patients with advanced malignancies at risk of developing SREs^6^. However, the potential duration of BP exposure is also likely to increase in this population, leading to an even higher prevalence of MRONJ. In Korea, the incidence of MRONJ in patients with oncologic conditions treated with intravenous BP ranges from 0 to 12,222 per 100,000 patient-years, and in osteoporosis patients prescribed BPs, it ranges from 1 to 90 per 100,000 patient-years^1^. The number of patients with osteoporosis and cancer is increasing as the population ages; naturally, the use of antiresorptive drugs for these patients is also increasing. However, the side effect called MRONJ is a significant medical burden when used as a treatment for patients with osteoporosis and cancer.

In MRONJ, jawbone exposure for > 8 weeks or fistula formation may lead to infection, which can cause an osteomyelitic lesion^3^. Some argued that infected MRONJ should be named medication-related osteomyelitis (OM) of the jaw and defined as an advanced condition to distinguish it from conventional OM, which can be effectively treated by antibiotics and surgical interventions^7,8^. However, studies have reported that infection is an essential event in MRONJ development^9^, and most histologic features obtained from MRONJ biopsy specimens exhibit the characteristics of classic OM^10^. Therefore, infection and OM could be accepted as a broad type of MRONJ^7,11^.

Recently, many researchers have attempted to describe imaging findings for the differentiation of MRONJ from conventional OM^11^ because MRONJ is refractory to antibiotic therapy and conservative debridement procedures. In addition, these lesions are challenging to treat, and their development in a patient with cancer may restrict medication usage and delay chemotherapy^12^. As part of this research series, we aimed to identify more characteristics of MRONJ that depended on the antiresorptive dosage. Only a few studies have described and compared MRONJ features between patients with osteoporosis and oncologic conditions^13^. Thus, this study aimed to investigate the clinical and radiological features of MRONJ in those two groups and clarify the differentiating aspects.

## Methods

### Patients

In this study, a retrospective chart and image review of 159 patients diagnosed with MRONJ was performed. The diagnostic criteria for MRONJ included a medical history of antiresorptive medication use confirmed through clinical, radiological, and histopathologic examinations at Seoul National University Dental Hospital from January 1, 2014, to December 31, 2018. The type, regimen, amount, and duration of antiresorptive drug therapy and bone exposure were not considered during the diagnostic process. Among the 159 patients included, 120 had been treated with antiresorptive drugs for osteoporosis management (osteoporotic group), and 39 had been treated with parenteral antiresorptive drugs to prevent SREs associated with cancer and bone metastasis (oncologic group). The oncologic group included 26 (66.7%) patients with breast carcinoma, 11 (28.2%) with multiple myeloma, 1 (2.6%) with prostate carcinoma, and 1 (2.6%) with renal cell carcinoma. Patients with a history of head/neck radiation therapy, orthognathic surgery, trauma, or bone metabolic disease other than osteoporosis were excluded.

Patients’ records, including medical/dental records and cone-beam computed tomography (CBCT) and/or multidetector row computed tomography (MDCT) and panoramic radiography images were collected. This study was exempted from review by the Seoul National University Hospital Institutional Review Board (ERI19020).

### Analysis of clinical features

General demographic information, including age and sex, was collected from electronic dental records (EDRs). Clinical signs and symptoms, symptom duration, and premedical histories, including the specific antiresorptive medication regimen and duration, and predental history were evaluated from the EDRs. The treatment method and prognosis written in the EDRs were logged. Treatment methods were divided into conservative (medications and dressing) and surgical (sequestrectomy, saucerization, and partial mandibulectomy). Lesion location was classified as follows: maxillary incisor, maxillary molar, mandibular incisor, mandibular molar, and retromolar trigone region. The staging of MRONJ according to the AAOMS position paper was adopted for classification^3^.

### Image analysis

Imaging features were analyzed on 159 panoramic radiographs and 159 CT images (148 MDCT and 11 CBCT). Panoramic images were obtained using OP 100 (Instrumentarium Dental, Tuusula, Finland). Most MDCT images were obtained using Somatom Sensation 10 (Siemens AG, Erlangen, Germany), and only 18 MDCT images were scanned using LightSpeed VCT (GE HealthCare, Milwaukee, Wisconsin, USA). Eleven CBCT images were obtained using Dinnova 3 (HDXwill Inc., Seoul, Korea). The radiologic findings were interpreted by consensus between two oral and maxillofacial radiologists with > 15 years of experience. The radiologists retrospectively interpreted the imaging findings using a picture archiving and communication system (Infinitt PACS, Infinitt Healthcare, Seoul, Korea).

**Figure 1:**
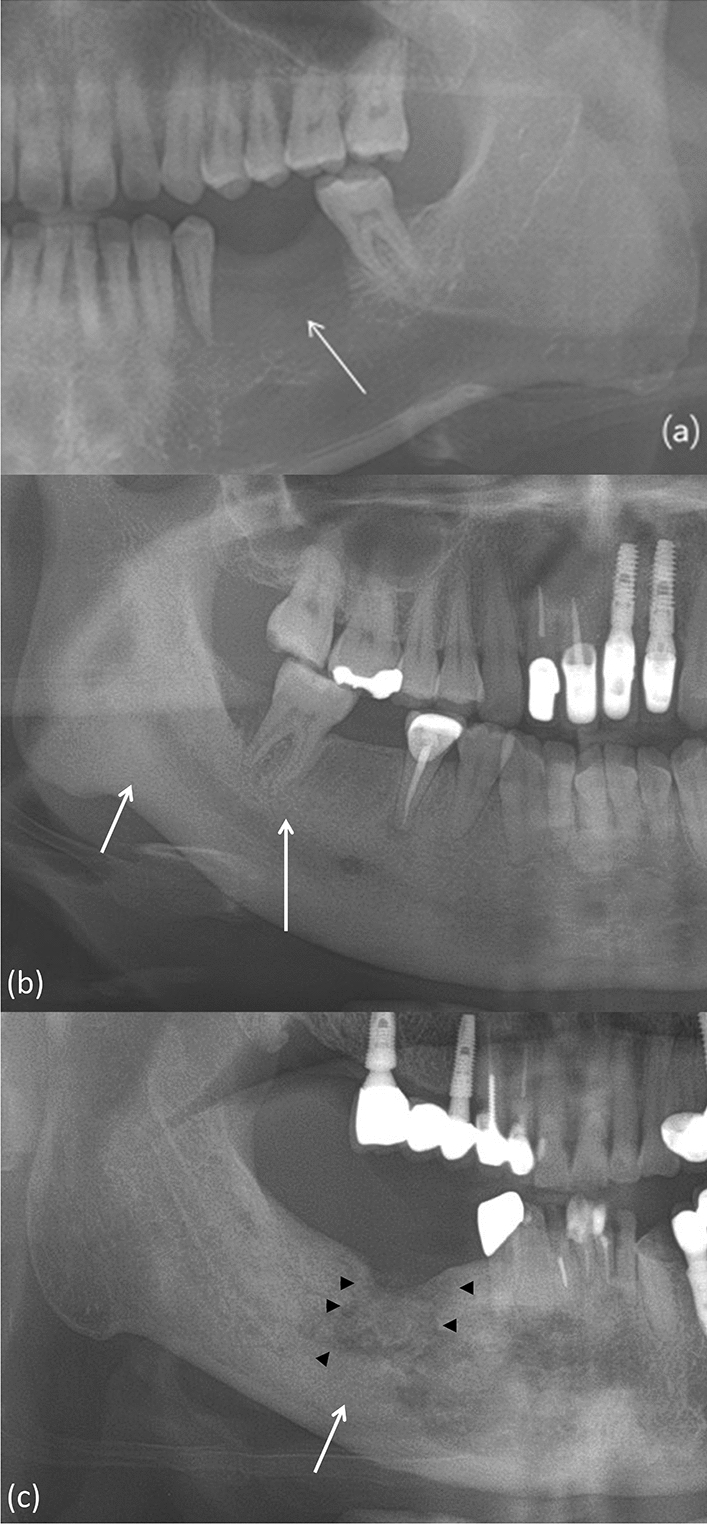
In panoramic radiographs, bone change patterns can be classified as (**a**) osteolytic bone change, as shown by the ill-defined radiolucency (white arrow) in the left premolar and molar region; (**b**) sclerotic bone changes, as indicated by the ill-defined diffuse radiopacity (white arrow) in the bone marrow; and (**c**) mixed osteolytic (black arrowheads) and sclerotic (white arrow) bone changes.

On panoramic radiographs, the lesion pattern was classified as osteolytic, sclerotic, or mixed (Fig. 1). The presence of sequestra, periosteal new bone formation, cortical bone involvement, and mandibular canal involvement was evaluated. The mandibular cortical index (MCI), a radiomorphometric analysis, was also evaluated and classified into one of three groups according to the method described by Klemetti et al.^14^.

**Figure 2:**
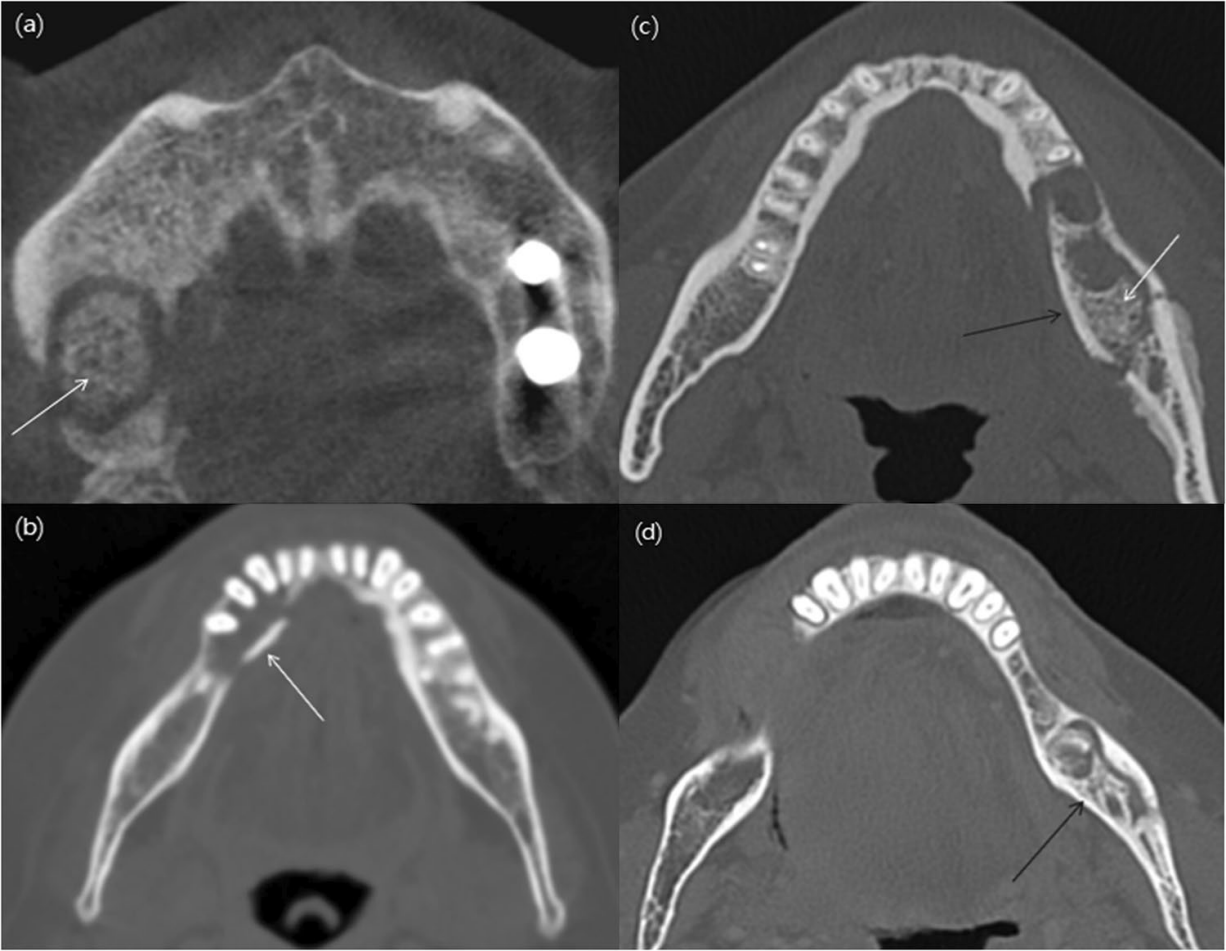
Analysis of hard tissue changes on computed tomography bone window images reveals various types of sequestra formation: (**a**) trabecular sequestrum; (**b**) cortical sequestrum; (**c**) mixed trabecular and cortical sequestrum, which includes both trabecular and cortical bones detached in a block; and (**d**) incomplete sequestrum, wherein part of the sequestrum is attached to the surrounding bone.

**Figure 3:**
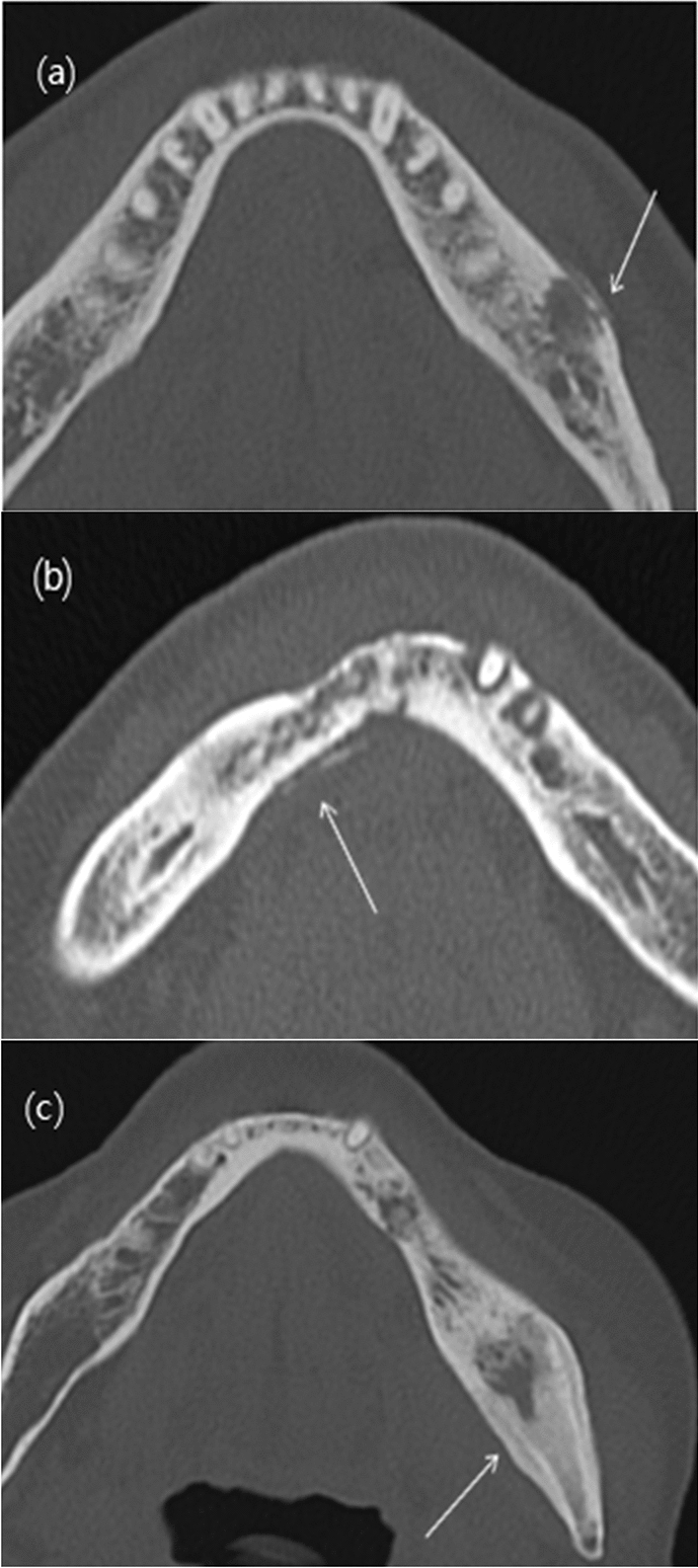
Patterns of periosteal new bone formation. Computed tomography images obtained with the bone window setting depict three types of periosteal new bone formation on the related cortical bone surface: (**a**) continuous lamellar, (**b**) interrupted lamellar, and (**c**) solid type.

Hard and soft tissue changes were analyzed on CT images. These hard tissue changes included the presence of trabecular and cortical defects, sclerosis, sequestrum, and periosteal new bone formation. The sequestra were classified as trabecular, cortical, mixed trabecular and cortical, or incomplete (Fig. 2). The size of each sequestrum was also measured. In addition, the periosteal new bone pattern was classified as continuous lamellar, interrupted lamellar, and solid (Fig. 3). CT images (except on 11 CBCT images) were evaluated for soft tissue changes to detect swelling, cellulitis, granulation tissue, sinusitis or mucositis, abscess, myositis, and fistula.

### Prognosis

For prognostic evaluation, patients were divided into cured and non-cured groups, and the demographic and clinical features were compared between the osteoporotic and oncologic groups. A cured case was defined as the presence of normal mucosal covering without exposed bone, absence of clinical signs of infection, and absence of active bone destruction visible on images 6 months after surgical intervention. The cure rates were evaluated in both the osteoporotic and oncologic groups.

### Statistical analysis

The clinical and imaging features were compared between the osteoporotic and oncologic MRONJ groups (n = 120 and 39, respectively). Statistical analyses were performed using IBM SPSS Statistics version 21.0 (IBM Corp., Armonk, NY, USA). All parameters are expressed as percentages. Student’s t-test was used to determine differences in continuous variables such as age, medication durations, and size of sequestra between the two groups. For categorical data, the chi-square test was used to indicate the difference in proportions in each group. Even when divided into multiple categories or stages, the chi-square test was used to determine whether a difference exists in the distribution between the oncologic and osteoporotic groups. A significance level of 5% was used in all tests.

### Ethical approval

This study was performed in accordance with the guidelines of the World Medical Association Helsinki Declaration for Biomedical Research involving Human Subjects and approved by the Institutional Review Board of Seoul National University Dental Hospital (ERI19020). Written or verbal informed consent was not obtained from any participants because the Institutional Review Board, waived the need for individual informed consent because this study had a non-interventional retrospective design and all data were analyzed anonymously.

## Results

This study analyzed data from a total of 159 subjects, including 17 (10.7%) men and 142 (89.38%) women. The average age was 72.0 (range, 41–91; women, 72.1; men, 70.7) years. In the osteoporotic group, 109 of 120 (90.8%) patients were treated with oral BPs, whereas 11 (9.2%) were treated with low-frequency BPs intravenously. In the oncologic group, 37 of 39 (94.9%) patients were treated with zoledronate intravenously, whereas the medications given in 2 (4.9%) patients could not be identified. The osteoporotic group was significantly older than the oncologic group, with less pronounced female predominance in the oncologic group. The average duration of antiresorptive drug therapy was longer in the osteoporotic group than in the oncologic group (*p* < 0.01), and the average symptom durations were 4.5 and 3.2 months in the osteoporotic and oncologic groups, respectively (Table 1). Most lesions occurred in the mandible, and the molar/premolar region was more affected. The maxillary and mandibular distributions of the lesions did not differ significantly between the two groups (Table 1).

**Table 1:** Demographic and clinical findings of medication-related osteomyelitis of the jaw (MROMJ) in the osteoporotic and oncologic groups.

	Osteoporotic group	Oncologic group	*P* value
Age (years)*	75.8 ± 7.2	60.4 ± 11.2	**0.000**
Sex (Male:Female) †	5:115	12:27	**0.000**
Medication duration (months)*	58.1 ± 47.6	28.0 ± 29.5	**0.000**
Symptom duration (months)*	4.5 ± 6.7	3.2 ± 4.3	0.299
Prognosis (Cured: Non-cured)†	57:29	10:20	**0.002**
Location (Mx : Mn) †	23 : 100	10:29	0.348
Maxilla- anterior (incisior)	4 (3.3%)	1 (2.6%)	
Maxilla-premolar/molar	19 (15.4)	9 (23.1%)	
Mandible- anterior (incisior)	14 (11.4%)	1 (2.6%)	
Mandible-premolar/molar	77 (62.6%)	24 (61.5%)	
Mandible—retromolar trigone	9 (7.3%)	4 (10.3%)	

Mx: maxilla; Mn: mandible.

* By T-test analysis.

^†^ By Pearson’s chi-square test.

Significant at *p* < 0.05.

Three cases in the osteoporosis group had lesions in both the Mx and Mn arches.

Prognosis was evaluated except for 43 patients who were not followed-up.

Significant values are in bold.

The staging and general clinical findings of the patients with MRONJ are shown in Table 2. Most lesions corresponded to MRONJ stages 2 and 3, and the distribution was not different between the osteoporotic and oncologic groups. The most frequent symptoms were pus discharge, pain, and swelling. Pus discharge and swelling were significantly more frequent in the osteoporotic group (*p* < 0.05), whereas bone exposure was significantly more prevalent in the oncologic group (*p* = 0.000).

**Table 2:** Staging and clinical signs, symptoms of the patients were compared between osteoporotic (n = 120) and oncologic (n = 39) group.

	Osteoporotic group (%)	Oncologic group (%)	*P* value
Staging*			0.147
Stage 0	0 (0.0)	1 (2.6)	
Stage 1	3 (2.5)	3 (7.7)	
Stage 2	93 (77.5)	28 (71.8)	
Stage 3	24 (20.0)	7 (17.9)	
Signs and symptoms			
Pain	64 (53.3)	25 (64.1)	0.239
Swelling	59 (49.2)	12 (30.8)	**0.010**
Pus discharge	75(62.5)	15 (38.5)	**0.004**
Numbness, paresthesia	12 (10.0)	1 (2.6)	0.351
Delayed healing	25 (20.8)	8 (20.5)	0.966
Mouth opening limitation	1 (0.8)	1 (2.6)	0.567
Bone exposure	24 (20.0)	21 (53.8)	**0.000**
Bleeding	16(13.3)	5 (12.8)	0.935
Fistula, intraoral	20 (16.7)	4 (10.3)	0.331
Fistula, extraoral	3 (2.5)	3 (7.7)	0.139
Tooth (or implant) mobility	5 (4.2)	4 (10.3)	0.382
Halitosis	4 (3.3)	3 (7.7)	0.249
Fracture	0 (0.0)	1 (2.6)	0.078

*Staging according to AAOMS MRONJ staging.

One patient may have multiple signs and symptoms.

By Pearson’s chi-square test.

Significant at *p* < 0.05.

Significant values are in bold.

**Figure 4:**
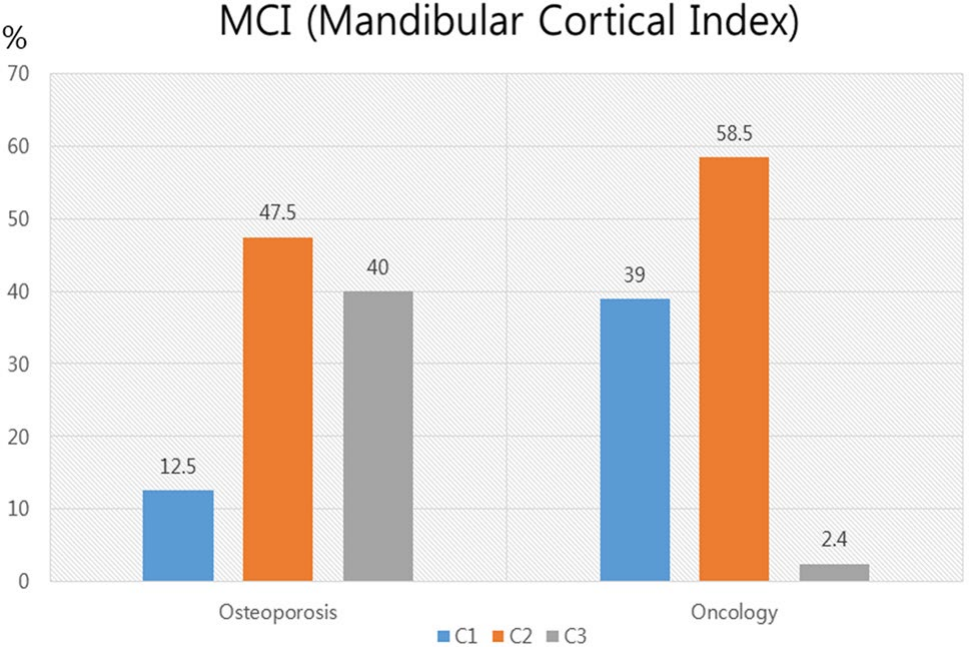
Mandibular cortical index (MCI: C1, C2, and C3) in the osteoporotic and oncologic groups.

A mixed pattern of sclerosis and osteolysis, followed by an osteolytic bone pattern, was the most common finding on panoramic radiographs (n = 159). The incidence rates of sequestrum, periosteal new bone, cortical bone involvement, and mandibular canal involvement were not statistically significant between the two groups (Table 3). The MCI was significantly higher in the osteoporotic group than in the oncologic group (*p* < 0.01). C3 and C1 were more frequently observed in the osteoporotic and oncologic groups, respectively (Fig. 4).

**Table 3:** Panoramic radiographic findings (159 patients).

	Osteoporotic group (%)	Oncologic group (%)	*P* value
Bone pattern of lesion			**0.027**
Mixed	89 (74.2)	28 (71.8)	
Osteolytic	18 (15.0)	1 (2.6)	
Sclerotic	3 (2.5)	1 (2.6)	
Not detected	10 (8.3)	9 (23.1)	
Sequestrum	56 (46.7)	14(35.9)	0.239
Periosteal new bone formation	4 (3.3)	2(5.1)	0.609
Cortical bone involvement	15(12.5)	7 (17.9)	0.140
Mandibular canal involvement†	43 (43.0)	9 (31.0)	0.247
Sclerosis	45 (37.5)	24 (61.5)	**0.009**

Significant at *p* < 0.05.

^†^Calculation was performed only in mandibular lesion (n = 129).

Significant values are in bold.

On CT images, the proportions of sequestra and periosteal new bone formation were comparable in both groups. Trabecular defect and sclerosis were the most frequent findings, followed by cortical defects and sequestra, and these findings did not differ significantly between the two groups (Table 4). The mean sequestra size was significantly larger in the oncologic group than in the osteoporotic group (15.3 vs. 10.6 mm, *p* < 0.05). Trabecular sequestrum was the most common type in the osteoporotic group, whereas mixed trabecular and cortical sequestra were the most frequent types in the oncologic group (*p* < 0.01). Incomplete sequestrum was also observed, as it remained partly attached to the surrounding bone. Of the 42 (35%) patients in the osteoporotic group and 16 (41.0%) in the oncologic group who exhibited periosteal new bone formation on CT, the lamellar type was the most frequent, followed by the solid type (Table 4). Soft tissue changes were observed in 81 (71.1%) patients in the osteoporotic group and 27 (77.1%) in the oncologic group, and swelling was the most frequent finding. Soft tissue changes did not differ significantly between the two groups (Table 4). Overall, the presence and severity of most radiographic findings did not differ significantly between the osteoporotic and oncologic groups, except for the MCI on panoramic radiographs and sequestrum on CT.

**Table 4:** Summary of imaging features using CT scans associated with medication-related osteomyelitis of the jaw (MROMJ; 159 patients) and the differences between osteoporotic group and oncologic group.

	Osteoporotic group (%)	Oncologic group (%)	*P* value
Hard tissue changes in CT images^†^			
Trabecular defect	107 (89.2)	34 (87.2)	0.734
Cortical defect	97 (80.8)	33 (84.6)	0.595
Sclerosis	105 (87.5)	34 (87.2)	0.958
Sequestrum	93 (77.5)	28 (71.8)	0.468
Periosteal new bone	42(35.0)	16 (41.0)	0.497
Presence of sequestrum			
Mean size of sequestrum*(mm)	10.6 ± 6.1	15.3 ± 11.1	**0.042**
Type of sequestrum on CT†(n = 121)			**0.000**
Trabecular	57 (61.3)	8 (28.6)	
Cortical	4 (4.3)	2 (7.1)	
Cortical + Trabecular	10 (10.8)	16 (57.1)	
Incomplete	22 (23.7)	2 (7.1)	
Presence of periosteal new bone formation			
Type of periosteal new bone on CT^†^ (n = 58)	.229		
Lamellar, continuous	26 (61.9)	9 (56.3)	
Lamellar, interrupted	14 (33.3)	4 (25.0)	
Solid	2 (4.8)	3 (18.8)	
Presence of soft tissue changes^†^	81 (71.1)	27 (77.1)	0.543
Type of Soft tissue changes in CT images (n = 148)			
Swelling	64 (56.6)	21 (60.0)	
Cellulitis	10 (8.8)	4 (11.4)	
Granulation tissue	15 (13.3)	3 (8.6)	
Sinusitis or Mucositis	1 (0.9)	3 (8.6)	
Abscess	7 (6.2)	3 (8.6)	
Myositis	1 (0.9)	0 (0.0)	
Fistula	2 (1.8)	1 (2.9)	

*By T-test analysis.

^**†**^By Pearson’s chi-squared test.

Significant at *P* < 0.05.

Significant values are in bold.

Most of the patients underwent surgery (osteoporotic group, 78.3%; oncologic group, 76.9%), and the rest received conservative therapy. The treatment choice did not differ between the osteoporotic and oncologic groups. In the comparison of prognosis, the cured rate was significantly higher in the osteoporotic group than in the oncologic group (66.3% vs 33.3%, *p* = 0.002). The cure rates were not different according to staging. The cured group in the osteoporotic group was significantly younger (*p* = 0.049). Although the cured patients in the oncologic group were older, this difference was not significant. Sex, symptom duration, or medication duration were not significantly different between the cured and non-cured groups.

## Discussion

In this study, the clinical and radiological features of MRONJ were explored in a relatively large number of cases and compared between the osteoporotic and oncologic groups. Clinically, in this study, patients with oncologic MRONJ are younger, were less female predominant, had shorter medication duration, and less cured. Although few significant radiological differences were found between the osteoporotic and oncologic groups, mandibular cortical bone change pattern on panoramic radiograph and sequestrum size and pattern on CT image were different.

Regarding demographic features, the osteoporotic group was older and predominantly female, which may be because older menopausal and postmenopausal women mainly use antiresorptive medications to treat and prevent osteoporosis. In a previous study, most MRONJ lesions affected the mandible in the oncologic group, whereas the maxilla and mandible were equally affected in the osteoporotic group^13^. Most previous studies of OM reported the predominant development of lesions in the mandible^15,16^, a region with a uniquely restricted blood supply; therefore, the antiangiogenic effects of BPs render the mandible more prone to avascular necrosis and OM^17^. Our observations in these groups confirmed the mandibular predominance of MRONJ lesions.

The incidence of MRONJ is significantly higher in patients on intravenous BP therapy than in those on oral BP therapy^6,18^. However, a study revealed that the discrepancy in the reported incidence of MRONJ among studies might be related to the differences in the inclusion criteria applied to the patients^19^. We identified 120 osteoporotic MRONJ cases and 39 oncologic MRONJ cases. Because of a limited number of patients in the oncologic group, the ratio of osteoporosis to patients with oncologic MRONJ differed significantly from those in previous studies^19^. In the present study, the average antiresorptive medication therapy was 58 months in the osteoporotic group and 28 months in the oncologic group. As the corresponding average symptom durations were 4.5 and 3.2 months, respectively, MRONJ might have occurred 54 and 24 months after low-dose and high-dose antiresorptive therapy. This result was quite similar to the findings of previous studies, which reported that this condition developed 12 months after intravenous BP injection and > 3 years after oral BP therapy^20,21^. In another study, the prevalence of MRONJ was greater among patients with > 4 years of exposure to oral BPs than in those with shorter durations of exposure (0.21% vs. 0.04%). Moreover, in that study, no MRONJ cases were found in a cohort of > 2000 cases with < 2.5 years of medication exposure^22^. However, in this study, we could identify several MRONJ cases with only 1–1.5 years of exposure to oral BP therapy. None of those cases were spontaneous, and all occurred after a suspected triggering dentoalveolar procedure, such as tooth extraction or dental implant placement, which is the most important precipitating cause of MRONJ^16^.

When patients with MRONJ were divided according to AAOMS MRONJ staging, most of the patients were classified as stage 2 or 3, and the distribution was not different between the osteoporotic and oncologic groups. Clinically, some signs and symptoms were different between the two groups, pus and swelling were more frequently observed in the osteoporotic group, and more than half of the patients showed bone exposure in the oncologic group. A previous study revealed a significant correlation between pus discharge and the lesion size delineated on CT, and lesions with a larger appearance on CT contained purulent secretion and sinus tracts^23^. However, if the lesion extent was thought to be determined by the sequestra size because the exact extent of the MRONJ lesion cannot be delineated, purulent signs were not significant in the oncologic group, which had a larger sequestrum. Instead, the oncologic group represented a larger sequestrum that includes the trabecular and cortical bone together.

In this study, we attempted to identify differences in imaging characteristics between the oncologic group that received high-dose antiresorptive therapy and the osteoporotic group that received lower-dose oral antiresorptive therapy. The CT findings of MRONJ in both groups were similar to those of conventional OM of the jaw^24^. Although no significant differences in radiographic signs were identified between the osteoporotic and oncologic groups, we observed typical image characteristics of advanced OM and significantly larger sequestra in the oncologic group. The MCI in panoramic radiograph was significantly higher in the osteoporotic group, which may reflect the initial underlying disease^14^. To the best of our knowledge, only a study explored the differences in the radiologic characteristics of MRONJ between the osteoporotic and oncologic groups^13^. In that study, the authors compared the composite radiographic index (CRI) according to the MRONJ stage. The low CRI group consisted of primarily patients with oncologic conditions, and CRI scores increased with MRONJ staging. Our results with larger and broader patterns of sequestrum in the oncologic group differ from those because this study is composed of patients with infected MRONJ mainly targeting stage 2 or 3.

In this study, the cure rates of this study were 66.3% group and 33.3% in the osteoporotic and oncologic groups, respectively. The rate was relatively lower than those in other studies that reported cured rates of 60–80%^25,26^. This difference was possibly due to the time point for determining cure set at 3 months, which is relatively shorter than that in other papers. According to Kaibuchi et al., the cured rate increased as the follow-up period increased^25^. Unlike other studies that calculated treatment rates differently for each treatment method, this study included both surgical and nonsurgical treatments, rather than separating them, which may have influenced these results. However, the lower cure rate in the oncologic MRONJ group was similar to those in other studies. The cured rate was significantly higher in the osteoporotic group than in the oncologic group. Considering clinical and radiological features, the oncologic MRONJ group had fewer purulent characteristics of infections such as pus or swelling than the osteoporotic MRONJ group; however, the bone is largely detached and exposed to the oral cavity. In the oncologic group, the inflammatory signs of pus discharge and sinus tracts might have been controlled to a certain extent, as the patients were already inpatients or were receiving concomitant drug therapy (e.g., corticosteroids). However, the recovery to healthy bones might be quite more difficult in the oncologic group because of high-dose antiresorptive agents and larger bone involvement. In this study, the findings, which can be summarized as a large form of osteonecrosis rather than purulent inflammatory changes and a low healing rate, suggest some precautions when using high-dose antiresorptive agents in patients with cancer. Oral hygiene should be checked before chemotherapy to prevent bone metastasis, and possible dental procedures must be considered, in advance which could increase the MRONJ risk. In addition, while taking antiresorptive medications, patients should be monitored for the occurrence of jaw necrosis, and oncologist work closely with the dentist to provide early treatment in early-stage MRONJ.

This study had some possible limitations. First, the oncologic group was not sufficiently large, and there might be statistical bias. This was a retrospective study, including all patients diagnosed with MRONJ who visited the dental hospital. The number of samples between the two groups is uneven, influenced by both the difference in the prevalence of osteoporosis and cancer and the difference in the incidence of MRONJ in the two groups. Despite the large difference in the sample size of the two groups, the sample size was not so small that confirming statistical significance was difficult. However, clinical interpretation might be challenging because of statistical bias due to differences in sample numbers. Second, obtaining exact information was challenging because data acquisition relied solely on EDRs. Third, this study did not explore the effects of factors such as the concurrent use of chemotherapy, other medications (e.g., glucocorticoids), or immunotherapy; presence of underlying comorbidities such as diabetes; or differences in BPs on MRONJ. Therefore, more controlled and prospective studies are required in the future to evaluate detailed differences according to antiresorptive medication dosage**.**

## Conclusions

This study demonstrated some significant features in the oncologic group, who had received high-dose antiresorptive therapy. These features included a more rapid disease onset, a symptom type (e.g., necrotic rather than purulent), and a low cure rate than those in the osteoporotic group. Although not many notable differences were found in the presence or severity of the radiologic pathognomonic features of MRONJ between the two groups, the sizes of sequestra and the MCI differed significantly between the osteoporotic and oncologic groups. Thus, these findings might aid the diagnosis, treatment planning, and prognostication of MRONJ in patients with osteoporosis and oncologic conditions in the future.

## Data Availability

All data and results analyzed during the current study are available from the corresponding author (noel1st@snu.ac.kr) upon reasonable request.

## Abbreviations

MRONJ: Medication-related osteonecrosis of the jaw
BP: Bisphosphonates
OM: Osteomyelitis
CBCT: Cone-beam computed tomography
MDCT: Multi-detector row computed tomography
MCI: Mandibular cortical index
